# Magnetic anisotropy control by applying an electric field to the side surface of ferromagnetic films

**DOI:** 10.1038/s41598-017-05799-8

**Published:** 2017-07-17

**Authors:** Hiroshi Terada, Shinobu Ohya, Le Duc Anh, Yoshihiro Iwasa, Masaaki Tanaka

**Affiliations:** 1 Department of Electrical Engineering and Information Systems, The University of Tokyo, 7-3-1 Hongo, Bunkyo-ku, Tokyo 113-8656 Japan; 2 Center for Spintronics Research Network, Graduate School of Engineering, The University of Tokyo, 7-3-1 Hongo, Bunkyo-ku, Tokyo 113-8656 Japan; 3 Institute of Engineering Innovation, Graduate School of Engineering, The University of Tokyo, 7-3-1 Hongo, Bunkyo-ku, Tokyo 113-8656 Japan; 4 QPEC and Department of Applied Physics, The University of Tokyo, 7-3-1 Hongo, Bunkyo-ku, Tokyo 113-8656 Japan; 5 RIKEN Center for Emergent Matter Science, Wako, 351-0198 Japan

## Abstract

Reducing the power consumption necessary for magnetization reversal is one of the most crucial issues facing spintronics devices. Electric field control of the magnetic anisotropy of ferromagnetic thin films is a promising method to solve this problem. However, the electric field is believed to be effective only within several nanometres of the surface in ferromagnetic metals because of its short Thomas-Fermi screening length, which prevents its practical application to devices. Herein, we successfully modulate the magnetic anisotropy of the *entire* region of the ferromagnetic layers in the elongated mesas of vertical spin field-effect transistors with widths as large as ~500 nm by applying an electric field to the *side surface* of the metallic GaMnAs-based mesas through an electric double layer. Our results will open up a new pathway for spintronics devices with ultra-low power consumption.

## Introduction

The electric field control of ferromagnetism is expected to be a key technique for future low-energy, non-volatile spintronics devices^1–4^. Recently, the Curie temperature and magnetic anisotropy of ferromagnetic thin films have been shown to be modulated by a gate electric field applied to the top surface of the films. However, the collective modulation of magnetic anisotropy is effective only when the film thickness is extremely thin (~a few nanometres)^1, 2^, so it is difficult to use this technique in practical applications. Thus, new techniques to overcome this problem are urgently needed.

**Figure 1:**
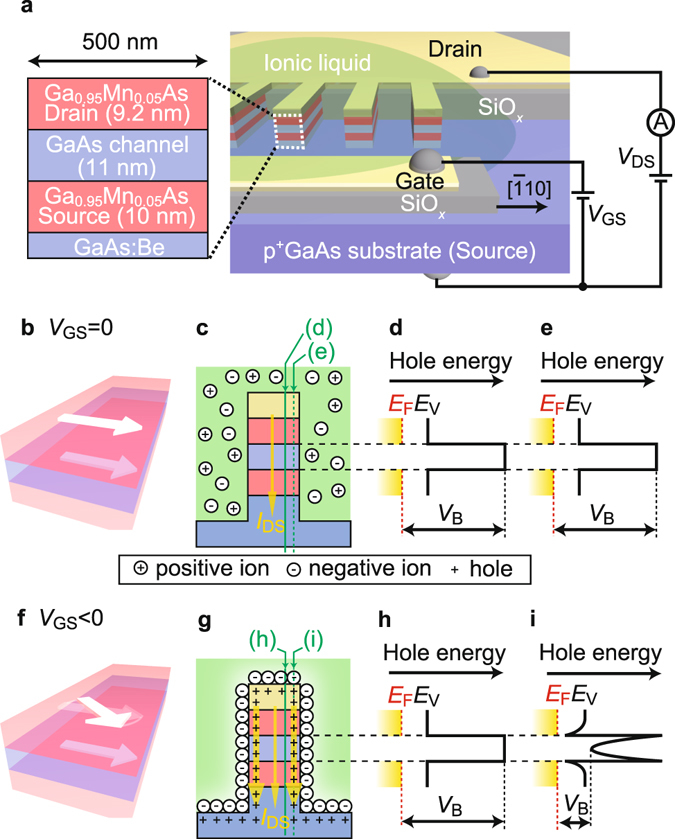
Vertical spin EDLT and its operation principle. (**a**) Schematic illustration of the vertical-spin EDLT with a thin GaAs channel (11 nm) between the ferromagnetic GaMnAs source and drain electrodes prepared and examined in this study. The comb-shaped drain electrode is connected to the top of the 30 elongated mesas, which have a width of 500 nm and a length of 50 μm. The substrate was used as the source electrode. A Au/Cr gate electrode was deposited on an insulating SiO_*x*_ film next to the mesas. The gate electrode and mesas were covered with electrolyte (DEME-TFSI). (**b**,**f**) Schematic illustrations of our findings regarding the modulation of the easy magnetization axis (white arrows) by the electric field. (**c**,**g**) Movement of the ions in the electrolyte. (**d**,**h**) Band alignment in most of the region inside the mesas (green solid lines in (**c**,**g**)). (**e**,**i**) Band alignment at several nanometres from the side surface of the mesas (green dotted lines in (**c**,**g**)). Here, (**b–e**) and (**f**–**i**) correspond to the cases without and with an electric field, respectively. In (**d**,**e**,**h**,**i**), the black solid lines represent the top of the valence band (*E*_V_), the red dotted lines are the Fermi level (*E*_F_) in GaMnAs, and the yellow regions represent holes. By varying the gate electric field, the effective barrier height *V*_B_ at the side surface changes, and *I*_DS_ (yellow arrows in (**c**,**g**)) is modulated.

The vertical spin electric-double-layer transistor (EDLT) used in our study (Fig. 1) is a type of a spin metal-oxide-semiconductor field-effect transistor (spin MOSFET)^5^, which has ferromagnetic source and drain electrodes and a semiconductor channel. Because of their current-amplification ability and compatibility with mature semiconductor technologies, spin MOSFETs have promising applications, such as low-power reconfigurable logic gates^6^ and non-volatile power gating^7^, with novel functions. In spin MOSFETs, a spin-polarized current is injected from the ferromagnetic electrode to the channel and controlled by the magnetization directions of the source and drain. Most of the previous studies were focused on conventional *planar* spin transistors^8–12^; however, the obtained magnetoresistance (MR) ratios were only 0.1%^8^, 0.005%^9^, and 0.03%^10^. This is because the carrier spins are quite sensitive to the crystal quality at the interfaces between ferromagnetic layers and semiconductors and because the channel length (~μm) is too long to prevent the influence of spin relaxation.

In this study, we adopt a *vertical* transistor structure^13^, which is more practical because it allows for the achievement of a short channel length on the order of nanometres with atomic-level control (Fig. 1a). Thus, we can significantly suppress the spin-relaxation during transport. In theory, we can realize large MR as high as that is obtained in magnetic tunnel junctions in the vertical device. We prepared elongated-mesa vertical EDLTs with a width of 500 nm. In EDLTs^14–17^, we can apply a large electric field of up to ~10 MV/cm to the side surface of the mesas using an ionic liquid. We successfully achieved a modulation ratio of the drain-source current *I*_DS_ up to ~20%, which is much larger than that obtained in the previous study on a vertical spin MOSFET (~0.5%)^13^, and we also obtained a large MR ratio (~5%) that was 50–1000 times as large as those observed in studies of conventional planar spin field-effect transistors^8–10^. Furthermore, we found that the magnetic anisotropy of the ferromagnetic layers is modulated by the electric field (Fig. 1b,f). Because the electric field penetrates only less than 10 nm into the GaMnAs layers^13^, and because the collective gate electric field modulations of ferromagnetism of GaMnAs were observed only in extremely thin films, this behaviour was completely unexpected in such wide and elongated mesas with a width of 500 nm. Our results indicate that applying an electric field to the side surface of the ferromagnetic layers may be useful for the collective control of ferromagnetism in spintronics devices.

## Results

### Samples

The device used in this study has a thin GaAs channel (11 nm) between the ferromagnetic Ga_0.95_Mn_0.05_As source and drain electrodes (Fig. 1a) (see the Methods section). The ferromagnetic p-type semiconductor GaMnAs is one of the most appropriate materials for a proof-of-concept study of a spin MOSFET because it can be epitaxially grown on a semiconductor GaAs without any dislocations. This can significantly suppress the spin scattering of the tunnelling carriers. We fabricated elongated mesas with a width of 500 nm and a length of 50 μm. The comb-shaped drain electrode is connected to the tops of the 30 mesas. The substrate was used as a source electrode. By applying a gate bias voltage *V*_GS_ to a Au/Cr gate electrode on an insulating SiO_*x*_ film next to the mesas, an electric field was applied to the side surface of the mesas through the electrolyte (N,N-diethyl-N-(2-methoxyethyl)-N-methylammonium bis(trifluoromethylsulfonyl-imide), [DEME-TFSI]). Thus, holes are accumulated or depleted at the side surfaces of the mesas by *V*_GS_, thereby modulating *I*_DS_ (Fig. 1b–i). Here we note that the electric field modulation of the band structure is more effective in the intermediate GaAs layer than in the top and bottom GaMnAs layers because there are many holes and thus strong screening in the GaMnAs layers. In the GaMnAs layers, the potential only at the side surface is slightly modulated. Thus, the electric lines of force originating from the side wall of the GaAs layer should go to one of the GaMnAs electrodes, which results in a vertical component of the electric field in GaAs (Fig. 1i). All the measurements were performed at 3.8 K, which is much lower than the Curie temperature (typically ~110–130 K when the Mn content is 6%^18^ of GaMnAs).

### Spin transistor operation

**Figure 2:**
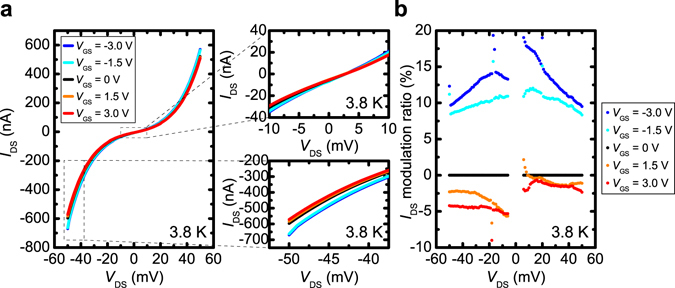
*I*_DS_-*V*_DS_ characteristics at various *V*_GS_. (**a**) *I*_DS_-*V*_DS_ characteristics measured at various *V*_GS_ ranging from −3.0 to 3.0 V at 3.8 K. (**b**) *I*_DS_ modulation ratios for various *V*_GS_ at 3.8 K.

As shown in Fig. 2a, plotting *I*_DS_ *vs*. the drain–source voltage *V*_DS_ reveals that |*I*_DS_| increases or decreases as *V*_GS_ decreases or increases, as expected. The modulation ratio of *I*_DS_, which is defined as [*I*_DS_ (*V*_GS_) − *I*_DS_ (*V*_GS_ = 0 V)]/*I*_DS_ (*V*_GS_ = 0 V), is 10–20% when *V*_GS_ is −3.0 V (Fig. 2b). The significant improvement of the modulation ratio relative to that observed in the previous study (~0.5%)^13^ is achieved by reducing the size of the device and increasing the electric field using the ionic liquid.

**Figure 3:**
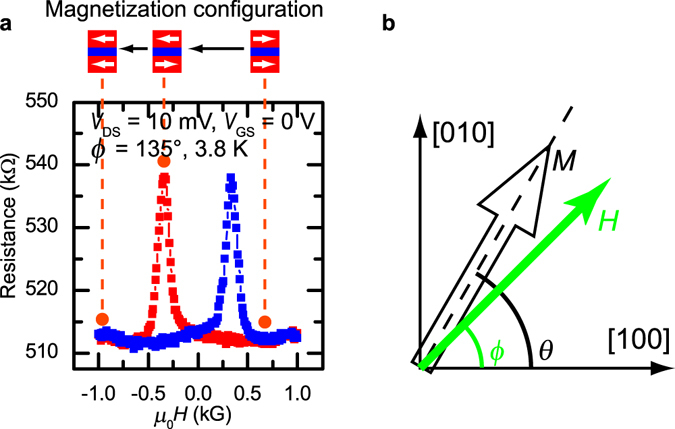
MR characteristics and definitions of angles *ϕ* and *θ*. (**a**) Tunnel resistance as a function of the magnetic field *μ*_0_ *H* at *V*_DS_ = 10 mV and *V*_GS_ = 0 V when *ϕ* = 135° (*H*//[$\bar{1}10$]) at 3.8 K. The tunnel resistance varies with the magnetization configuration of the two GaMnAs layers. (**b**) Definitions of angles *ϕ* of *H* and *θ* of *M* in the (001) film plane.

For spin transistor operation, the *I*_DS_ modulation by the magnetization alignment is important. As shown in Fig. 3a, the tunnel resistance is controlled by the magnetization configuration of the two GaMnAs layers; the tunnel resistance is high in the anti-parallel magnetization configuration and low in the parallel one. It is noteworthy that the anti-parallel magnetization configuration is realized because the coercivities of top and bottom GaMnAs layers are different due to the heating during the growth^19^. Here, the MR ratio is defined as (*R*_AP_ −* R*_P_)/*R*_P_, where *R*_P_ and *R*_AP_ are the tunnel resistances in the parallel and anti-parallel configurations, respectively. Therefore, in our vertical spin EDLT, current modulation by both the gate electric field and the magnetization alignment is successfully achieved.

### Magnetic anisotropy control

**Figure 4:**
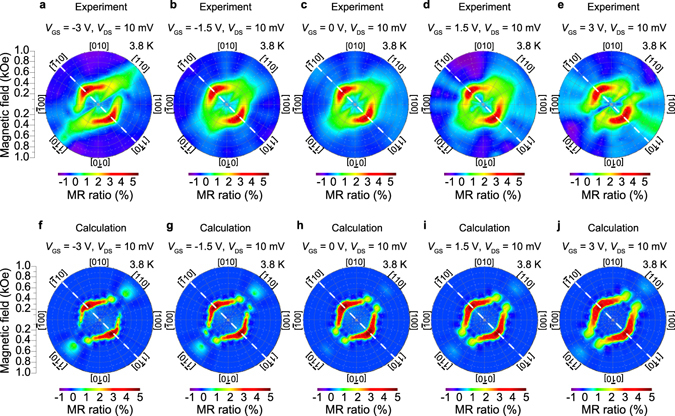
Experimental and calculated *ϕ* dependences of the MR at various *V*_GS_. (**a**–**e**) Experimentally obtained MR characteristics as a function of the magnetic field direction *ϕ* at each *V*_GS_ when *V*_DS_ = 10 mV at 3.8 K. For each *ϕ*, we applied a strong magnetic field in the opposite direction of *ϕ* and decreased it to zero before the measurements. Subsequently, we measured the tunnel resistance while increasing the magnetic field in the *ϕ* direction. The measurements were carried out using a step size of 15° of *ϕ*. (**f**–**j**) Calculated MR characteristics as a function of *ϕ* using the coherent rotation model. The magnetic anisotropy fields used in the calculations are summarized in Fig. 5a,b. Here, *P* in equation (2) was set to 0.15.

Furthermore, we found that the electric field applied to the side surface of the device influences the magnetic anisotropy of the entire region of the GaMnAs layers in these elongated mesas. The magnetic anisotropy was investigated by measuring the in-plane magnetic-field direction *ϕ* dependence of the MR (Fig. 4a–e). Here, *ϕ* is defined as the angle from the in-plane [100] direction (Fig. 3b), and the MR ratio is defined as [*R*(*H*) − *R*(0)]/*R*(0) at each *ϕ*, where *R*(*H*) is the tunnel resistance when the applied magnetic field is *H*. The observed anisotropy of MR means that the obtained MR characteristics are originated from tunnel magnetoresistance effects, not from tunnel anisotropic magnetoresistance effects (For the detail, see Supplementary Note 1, and Supplementary Fig. S1). As shown in Fig. 4a–e, both of the MR patterns and the MR ratio are modulated with *V*_GS_. When *V*_GS_ = 0 V, the MR pattern is symmetric with respect to the [$\bar{1}10$] axis (Fig. 4c). Meanwhile, when *V*_GS_ < 0 V, it becomes asymmetric (Fig. 4a); the red-coloured regions, which correspond to the nearly ideal anti-parallel magnetization configuration, extend in the [110] (or [$\bar{1}\bar{1}0$]) direction. When *V*_GS_ > 0 V, the red-coloured regions are broadened, and the pattern has a slight tendency to be four-fold symmetric along the 〈100〉 direction (Fig. 4e).

## Discussion

To understand the mechanism underlying this behaviour, we calculated and reproduced the polar plot of the MR ratio using the Stoner-Wohlfarth coherent rotation model. In this model, the magnetostatic energy *E* of a ferromagnetic film, which has a magnetization *M* in the in-plane direction of *θ* with respect to the in-plane [100] direction (Fig. 3b), is expressed by^3, 20^

1 $$\begin{array}{rcl}E & = & \frac{M{H}_{{\rm{B}}}}{8}{\sin }^{2}(2\theta )+\frac{M{H}_{{\rm{U}}[\bar{1}10]}}{2}{\sin }^{2}(\theta -\frac{3\pi }{4})\\  &  & +\frac{M{H}_{{\rm{U}}[010]}}{2}{\sin }^{2}(\theta -\frac{\pi }{2})-MH\,\cos (\theta -\varphi ),\end{array}$$

where *H*_B_, ${H}_{U[\bar{1}10]}$ and *H*_U[010]_ are the biaxial anisotropy field along 〈100〉 (*i*.*e*., [100] and [010]), the uniaxial anisotropy field along [$\bar{1}10$] and that along [010], respectively. In this model, *θ* is determined so that *E* takes a local minimum. In addition, we allowed the magnetization to be reversed by the nucleation and propagation of the 180° domain wall; in this case, *θ* moves from one local minimum to another local minimum with a lower energy when the energy difference between these points exceeds the domain nucleation/propagation energy *ε*. When the relative angle of the magnetization directions of the top and bottom ferromagnetic layers in our device is Δ*θ*, the MR ratio is expressed by^21^

2 $$(\text{MR}\,\text{ratio})=\frac{{P}^{2}(1-\,\cos ({\rm{\Delta }}\theta ))}{1+{P}^{2}\,\cos ({\rm{\Delta }}\theta )},$$

where *P* is the spin polarization of the ferromagnetic layers.

**Figure 5:**
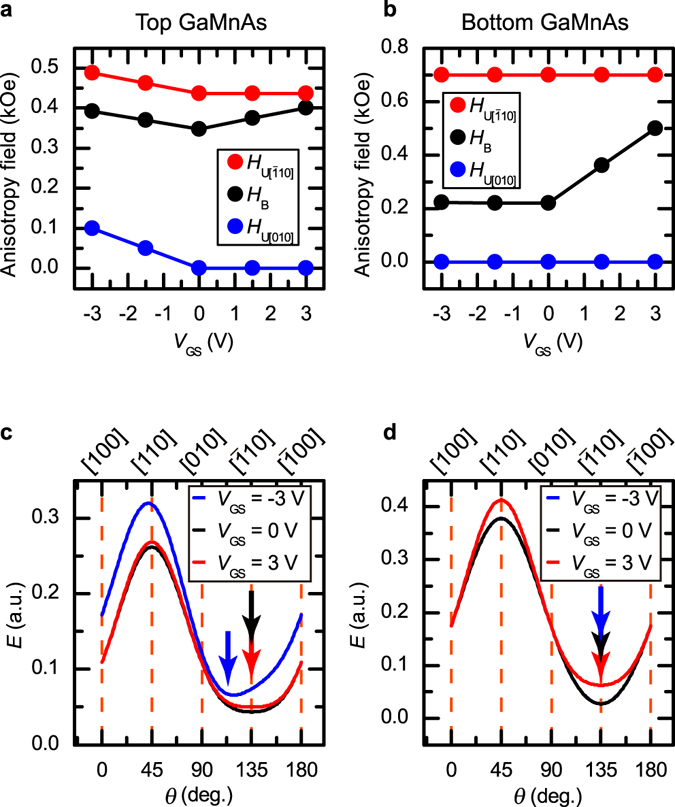
Derived magnetic anisotropy fields of the GaMnAs layers at various *V*_GS_. (**a**,**b**) Obtained magnetic anisotropy fields *H*_B_, ${H}_{U[\bar{1}10]}$ and ${H}_{U[010]}$ as a function of *V*_GS_ for the top GaMnAs layer (**a**) and the bottom GaMnAs layer (**b**). The values of *ε*/*M* for the top and bottom GaMnAs layers were set to 0.568 and 0.830, respectively. (**c**,**d**) Calculated magnetostatic energy *E* at *H* = 0 as a function of the magnetization direction *θ* for the top GaMnAs layer (**c**) and the bottom GaMnAs layer (**d**) when *V*_GS_ = −3 V, 0 V, and 3 V obtained with the magnetic anisotropy fields shown in Fig. 5a,b. Magnetic easy axes corresponding to the local minima of *E* are indicated by arrows.

Using equations (1) and (2) and the values of *H*_B_, ${H}_{U[\bar{1}10]}$ and *H*_U[010]_ plotted in Fig. 5a,b, we can well reproduce the *ϕ* dependence of the MR (Fig. 4f–j, see the Methods section, Supplementary Note 2, and Supplementary Fig. S2). The difference in the *V*_GS_ dependence of the anisotropy fields between the top and bottom GaMnAs layers probably results from the different quality between the two layers. As shown in Fig. 5a,b, the uniaxial anisotropy along [$\bar{1}10$] (${H}_{U[\bar{1}10]}$) is dominant in both GaMnAs layers. As *V*_GS_ increases, the biaxial anisotropy (*H*_B_) tends to be stronger. This tendency is different from that in the previous report, in which the uniaxial anisotropy increased as the gate electric field applied to the top surface of the GaMnAs layers increased^1^. This difference may be caused by the difference in the electric field direction (*i*.*e*., parallel to the side surface *vs*. parallel to the top surface) or the film quality.

In our model, domain walls are considered only when the magnetization switch occurs. Thus, in principle, our model is a single-domain model. Therefore, the good agreement between the experimental *ϕ* dependence of the MR (Fig. 4a–e) and that calculated using this model (Fig. 4f–j) indicates that the magnetic anisotropy of the *entire* region of the elongated mesas (width: 500 nm) is changed by the gate electric field applied to the side surface (Fig. 5a–d). In particular, as shown in Fig. 5c, the magnetic easy axis of the top GaMnAs layer is rotated by 16° (Fig. 1b,f), which is larger than that (~10°) observed in the previous study^1^, by the gate electric field. This behaviour is surprising and cannot be explained in the current understanding of the electric field’s control of ferromagnetism, because magnetic anisotropy is modulated in such wide elongated mesas (~500 nm), while the potential is modulated only within ~10 nm of the side surface of the GaMnAs layers. This situation may be similar to the case of the collective metal-insulator transition observed in VO_2_ induced by the gate electric field applied to the film’s top surface^22^. In ref. 22, the metal-insulator transition was observed in the entire region of films with thicknesses up to 70 nm, although the charges were accumulated only at the top surface of the films. The collective phase transition occurs because it is favourable to remain in a single phase at length scales less than a domain size to minimize interface energy^22^. In the analogy of the metal-insulator transition in VO_2_, the collective modulation of ferromagnetism in GaMnAs can be explained. In GaMnAs, the typical magnetic domain size is a few μm^23^, and it may not be energetically favourable for a domain wall to form along the longitudinal direction of the elongated mesas. Thus collective control of the magnetic anisotropy is possible just by changing the magnetic properties within 10 nm of the side surface.

Our results indicate that applying an electric field to the side surface of the ferromagnetic layers may be quite effective for the manipulation of magnetization in spintronics devices, and suggest that the shape or size of the mesas is important for the collective control of the ferromagnetism in thin films. Further miniaturization and exploring more appropriate materials will realize the more effective modulation even at room temperature.

## Methods

### Growth

The heterostructure composed of Ga_0.95_Mn_0.05_As (9.2 nm)/GaAs (11 nm)/Ga_0.95_Mn_0.05_As (10 nm)/GaAs:Be (100 nm) was grown on a p^+^GaAs (001) substrate by low-temperature molecular beam epitaxy. The substrate temperatures during the growth of the bottom Ga_0.95_Mn_0.05_As, the middle GaAs, and the top Ga_0.95_Mn_0.05_As layers were 205 °C, 185 °C and 195 °C, respectively.

### Process

After the growth, we fabricated the vertical spin EDLT structure. We partially etched the grown films, buried the etched area under a SiO_*x*_ insulating film, and then formed a contact pad for the drain electrode on the insulating film to allow *I*_DS_ to flow only in the patterned mesa diodes, which have a width of 500 nm and a length of 50 μm. In our measurements, before changing *V*_GS_, we increased the sample temperature to 220 K from the measurement temperature of 3.8 K because, at this temperature, the ionic liquid is frozen and the ions in the electrolyte cannot move. After changing *V*_GS_, the sample was cooled to 3.8 K while maintaining *V*_GS_. Although the Au/Cr drain electrode and the GaAs:Be layer are also covered by the ionic liquid, they are metallic and thus the gate-modulation effect in them are negligibly small (see Supplementary Note 3, and Supplementary Fig. S3).

### Measurements

The *I*_DS_-*V*_DS_ characteristics were measured before *H* was applied at each *V*_GS_. Thus the magnetization direction of every magnetic domain in each GaMnAs layer was not aligned in the same direction, because the sample temperature was increased to ~220 K, which is much higher than the Curie temperature (typically ~110–130 K when the Mn content is 6%) of the GaMnAs layers, to change *V*_GS_ before the *I*_DS_ - *V*_DS_ measurements. To measure the *ϕ* dependence of the MR, we applied a strong magnetic field of 1 T in the opposite direction of *ϕ* to first align the magnetization directions, and then we decreased the magnetic field to zero before the measurements. Subsequently, we started to measure the tunnel resistance while increasing the magnetic field from zero in the *ϕ* direction. The measurements were performed at every 15° step of *ϕ*. To calculate the *ϕ* dependence of the MR, *P* was set to 0.15. During the measurements of the *ϕ* dependence of the MR, the Joule heating was negligibly small (see the Supplementary Note 4, and Supplementary Fig. S4).

## Electronic supplementary material

Supplementary Information

## References

[1] Chiba D (2008). Magnetization vector manipulation by electric fields. Nature.

[2] Sawicki M (2010). Experimental probing of the interplay between ferromagnetism and localization in (Ga, Mn)As. Nature Physics.

[3] Chiba D (2011). Electrical control of the ferromagnetic phase transition in cobalt at room temperature. Nature Materials.

[4] Anh LD, Hai PN, Kasahara Y, Iwasa Y, Tanaka M (2015). Modulation of ferromagnetism in (In, Fe)As quantum wells via electrically controlled deformation of the electron wave functions. Phys. Rev. B.

[5] Sugahara S, Tanaka M (2004). A spin metal-oxide-semiconductor field-effect transistor using half-metallic-ferromagnet contacts for the source and drain. Appl. Phys. Lett..

[6] Matsuno T, Sugahara S, Tanaka M (2004). Novel Reconfigurable Logic Gates Using Spin Metal–Oxide–Semiconductor Field-Effect Transistors. Jpn. J. Appl. Phys..

[7] Shuto Y, Yamamoto S, Sugahara S (2009). Nonvolatile static random access memory based on spin-transistor architecture. J. Appl. Phys..

[8] Nakane R, Harada T, Sugiura K, Tanaka M (2010). Magnetoresistance of a Spin Metal-Oxide-Semiconductor Field-Effect Transistor with Ferromagnetic MnAs Source and Drain Contacts. Jpn. J. Appl. Phys..

[9] Sasaki T (2014). Spin Transport in Nondegenerate Si with a Spin MOSFET Structure at Room Temperature. Phys. Rev. Applied.

[10] Tahara T (2015). Room-temperature operation of Si spin MOSFET with high on/off spin signal ratio. Appl. Phys. Express.

[11] Koo HC (2009). Control of Spin Precession in a Spin-Injected Field Effect Transistor. Science.

[12] Chuang P (2015). All-electric all-semiconductor spin field-effect transistors. Nature Nanotechnology.

[13] Kanaki T, Asahara H, Ohya S, Tanaka M (2015). Spin-dependent transport properties of a GaMnAs-based vertical spin metal-oxide-semiconductor field-effect transistor structure. Appl. Phys. Lett..

[14] Saito Y, Iwasa Y (2015). Ambipolar Insulator-to-Metal Transition in Black Phosphorus by Ionic-Liquid Gating. ACS Nano.

[15] Saito Y, Kasahara Y, Ye J, Iwasa Y, Nojima T (2015). Metallic ground state in an ion-gated two-dimensional superconductor. Science.

[16] Shimotani H (2007). Insulator-to-metal transition in ZnO by electric double layer gating. Appl. Phys. Lett..

[17] Yamada Y (2011). Electrically Induced Ferromagnetism at Room Temperature in Cobalt-Doped Titanium Dioxide. Science.

[18] Muneta I, Ohya S, Terada H, Tanaka M (2016). Sudden restoration of the band ordering associated with the ferromagnetic phase transition in a semiconductor. Nature Communications.

[19] Ohya S, Muneta I, Hai PN, Tanaka M (2009). GaMnAs-based magnetic tunnel junctions with an AlMnAs barrier. Appl. Phys. Lett..

[20] Pappert K (2007). Detailed transport investigation of the magnetic anisotropy of (Ga, Mn)As. New Journal of Physics.

[21] Higo Y, Shimizu H, Tanaka M (2001). Anisotropic Tunneling Magnetoresistance in GaMnAs/AlAs/GaMnAs Ferromagnetic Semiconductor Tunnel Junctions. J. Appl. Phys..

[22] Nakano, M. *et al*. Collective bulk carrier delocalization driven by electrostatic surface charge accumulation. *Nature* **487**, 459 (2012). 10.1038/nature11296 22837001

[23] Pross A (2004). Magnetic domain imaging of ferromagnetic GaMnAs films. J. Appl. Phys..

