# Supplementary material for: Magnetic anisotropy control by applying an electric field to the side surface of ferromagnetic films
Source: Sci Rep. 2017 Jul 17;7:5618. doi: 10.1038/s41598-017-05799-8 (PMC5514098; doi:10.1038/s41598-017-05799-8)
Supplement: Supplementary file 1 — Supplementary Information [file 41598_2017_5799_MOESM1_ESM.pdf]

Supplementary Information

Magnetic anisotropy control by applying an electric field to the side surface of ferromagnetic films

Hiroshi Terada[1], Shinobu Ohya[1,2,3], Le Duc Anh[1,3], Yoshihiro Iwasa[4,5], and Masaaki Tanaka[1,2*]

1. *Department of Electrical Engineering and Information Systems, The University of Tokyo, 7-3-1 Hongo, Bunkyo-ku, Tokyo 113-8656, Japan*
2. *Center for Spintronics Research Network, Graduate School of Engineering, The University of Tokyo, 7-3-1 Hongo, Bunkyo-ku, Tokyo 113-8656, Japan*
3. *Institute of Engineering Innovation, Graduate School of Engineering, The University of Tokyo, 7-3-1 Hongo, Bunkyo-ku, Tokyo, 113-8656, Japan*
4. *QPEC and Department of Applied Physics, The University of Tokyo, 7-3-1 Hongo, Bunkyo-ku, Tokyo 113-8656, Japan*
5. *RIKEN Center for Emergent Matter Science, Wako 351-0198, Japan*

Supplementary Note 1.   TMR and TAMR characteristics

In order to verify that the MR characteristics obtained in the main manuscript originates from TMR rather than TAMR, we measured TAMR in a GaMnAs-based heterostructure that has a single ferromagnetic layer. We have grown $Ga_{0.94}Mn_{0.06}As$ (20 nm)/ AlAs (6 nm)/ GaAs: Be (100 nm) on a p^+ GaAs (001) substrate, and have prepared a cylindrical mesa diode with 200 μm in diameter as shown in Supplementary Fig. S1(a). We measured the MR varying the in-plane magnetic-field direction ϕ at V = -80 mV at 3.8 K. The ϕ dependence of the MR is measured in the same procedure as we performed in the main manuscript. Supplementary Fig. S1(c) shows the MR characteristics measured at ϕ = 35°, and 75°. When ϕ = 75°, the MR characteristics showed positive hysteresis signals; however, when ϕ = 35°, the MR showed negative hysteresis signals, which are not observed in the usual TMR characteristics regardless of ϕ. These are TAMR characteristics and are summarized in Supplementary Fig. S1(e). The obtained TAMR (0.2%) is negligibly small in comparison with the value of the TMR ratio (~5%) shown in the main manuscript. In order to understand these MR characteristics, we calculated the TAMR characteristics of a tunnel junction with a ferromagnetic GaMnAs electrode whose easy axis is in the $[\bar{1}10]$ direction. In the calculation, we used the Stoner-Wohlfarth model in the same way as we did in the main manuscript. Here, we assumed H_B = 0.1 (kOe), $H_{U[010]}$ = 0 (kOe), $H_{U[\bar{1}10]}$ = 0.5 (kOe), and

ε/M = 0.2 (kOe), where H_B, $H_{U[010]}$, $H_{U[\bar{1}10]}$, ε, and M are the biaxial anisotropy field along <100>, the uniaxial anisotropy field along [010], the uniaxial anisotropy field along $[\bar{1}10]$, the domain nucleation/propagation energy, and the magnetization, respectively.

In this case, using the concept of TAMR, the tunnel resistance R can be expressed by

$$R(\theta) = R_0 + \Delta R \cdot \cos(2\theta), \tag{S1}$$

where, R_0 and ΔR are fitting parameters. θ is the in-plane magnetization direction with respect to the [100] direction in GaMnAs. Then, the TAMR ratio is expressed by

$$\text{TAMR ratio} = \frac{R\big(\theta(H,\phi)\big) - R\big(\theta(0,\phi)\big)}{R\big(\theta(0,\phi)\big)}, \tag{S2}$$

where, $\theta(H, \phi)$ means θ under magnetic field H in the direction of ϕ. Supplementary Fig. S1(g) shows the calculated TAMR ratio. Our calculated result (Supplementary Fig. S1(g)) well explains the experimental result (Supplementary Fig. S1(e)), which indicates that the MR characteristics shown in Fig. S1(e) is originated from the TAMR effect.

Now, we compare the TAMR characteristics with the TMR characteristics obtained in the main manuscript. Supplementary Fig. S1(b) shows the schematic illustration of the GaMnAs-based device composed of $Ga_{0.95}Mn_{0.05}As$ (9.2 nm)/ GaAs (11 nm)/ $Ga_{0.95}Mn_{0.05}As$ (10 nm)/ GaAs:Be (100 nm) presented in the main manuscript. Here, we omit the gate electrode and electrolyte in the illustration. This device shows positive spin-valve characteristics at all ϕ as shown in Supplementary Figs. S1(d), and S1(f). The experimental and calculated MR characteristics as functions of ϕ are summarized in Supplementary Fig. S1(f), and S1(h), respectively. As shown in Supplementary Fig. S1(e) and S1(f), the TMR characteristics obtained in the main manuscript are completely different from TAMR characteristics, thus, we can conclude that the MR shown in our study originates from TMR, not from TAMR.

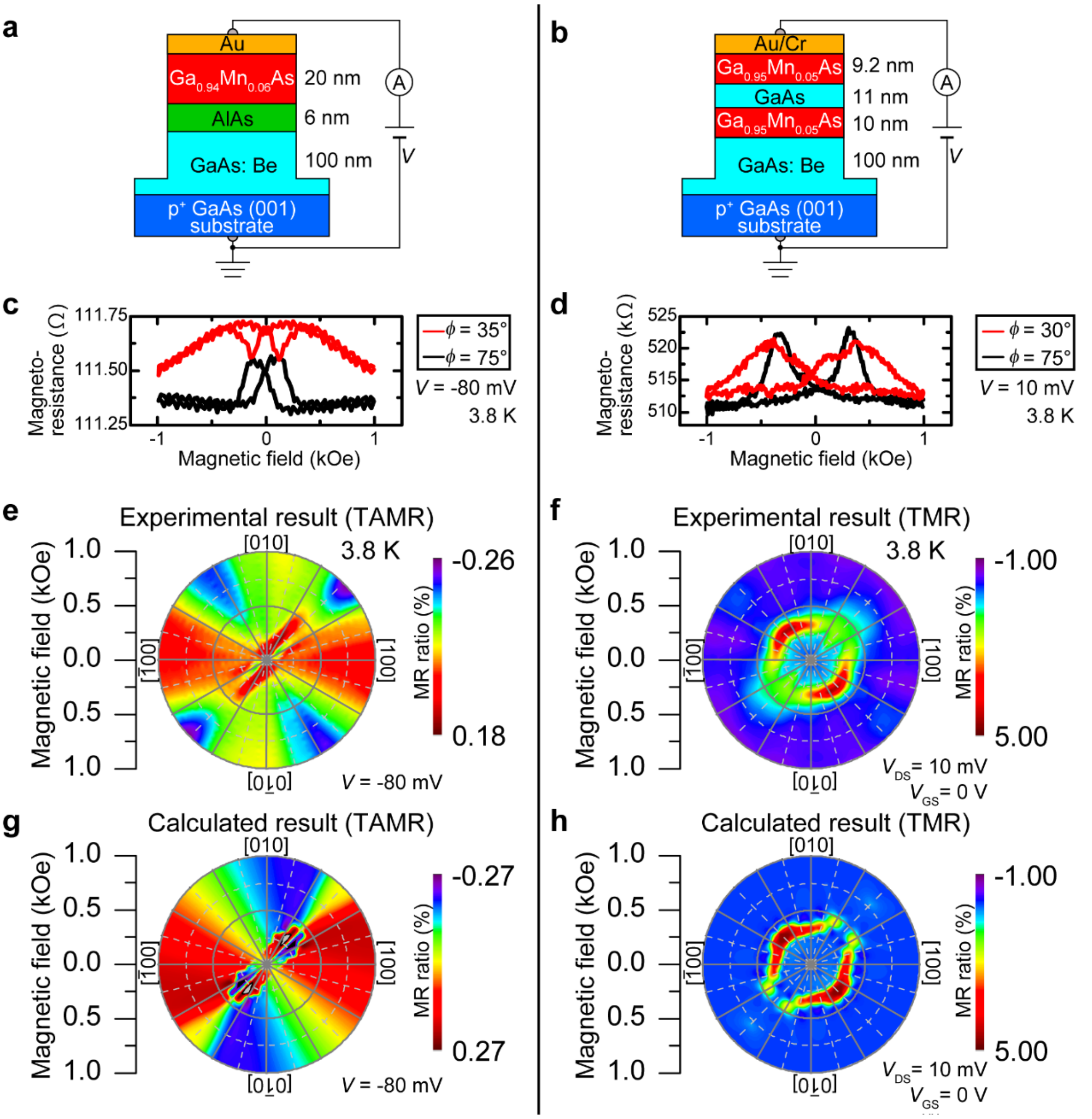

Supplementary Figure S1 | Comparison between TAMR and TMR characteristics. (a) Schematic illustration of the diode device for TAMR measurements. The diode is composed of $Ga_{0.94}Mn_{0.06}As$ (20 nm)/ AlAs (6 nm)/ GaAs: Be (100 nm) on a p^+ GaAs (001) substrate. (b) Schematic illustration of the GaMnAs-based device presented in the main manuscript, which is composed of $Ga_{0.95}Mn_{0.05}As$ (9.2 nm)/ GaAs (11 nm)/ $Ga_{0.95}Mn_{0.05}As$ (10 nm)/ GaAs: Be (100 nm) on a p+ GaAs (001) substrate. Here, we omit the gate electrode and electrolyte in the illustration. (c) MR characteristics obtained in the diode shown in Supplementary Fig. S1(a) at V = -80 mV, when ϕ = 35° and 75°. Both of the positive and negative hysteresis signals are observed. (d) MR characteristics obtained with the device shown in Supplementary Fig. S1(b) at V = 10 mV, when ϕ = 30° and 75°. MR characteristics show positive spin-valve characteristics at all ϕ. (e) Color contour plot of the MR characteristics as a function of ϕ obtained in the diode shown in Supplementary Fig. S1(a). (f) Color contour plot of the MR characteristics as a function of ϕ obtained in the device shown in Supplementary Fig. S1(b). (g) Color contour plot of the calculated TAMR characteristics as a function of ϕ. (h) Color contour plot of the calculated TMR characteristics as a function of ϕ.

Supplementary Note 2. Fitting routine of the anisotropy of magnetoresistance

In this study, we calculated the magnetic anisotropy of the two GaMnAs layers in our device by using the Stoner-Wohlfarth model, and derived the relative magnetization directions $\Delta\theta$ at every magnetic field H and magnetic field direction ϕ to obtain the ϕ dependence of the MR characteristics. We determined the anisotropy fields (H_B, $H_{U[010]}$, and $H_{U[\bar{1}10]}$) and domain nucleation/propagation energy ε so that we can reproduce the peak position and the MR ratio at all ϕ, simultaneously. Although it is difficult to completely reproduce the MR characteristics because the Stoner-Wohlfarth model cannot reproduce the gradual variation of magnetization M and θ, we can reproduce the characteristic anisotropy of the MR with this model.

Supplementary Fig. S2a-d shows the experimentally obtained MR characteristics at V_G = -3 V (thick black curves), and calculated MR characteristics (thin colored curves) when H_B, $H_{U[\bar{1}10]}$, $H_{U[010]}$, and ε/M of the top GaMnAs layer are varied, respectively, while parameters except for the varied one are fixed at the values used in the main manuscript in each graph. The peak positions of calculated result shown by red curves reproduce that of experimentally obtained results. Although, when we fit the MR characteristics at fixed ϕ, we can find some sets of parameters, when we fit the MR characteristics at all ϕ simultaneously, the parameters are determined almost uniquely.

We can determine the fitting parameters for top and bottom GaMnAs layers rather independently. Generally, the top GaMnAs layer has a smaller coercive field than the bottom layer. This is because the Mn interstitial defects can be more easily diffused out from the top GaMnAs to the surface than from the bottom layer during the growth [D. Chiba *et al.*, APL **82**, 3020 (2003).]. Thus, in our calculation, we adjusted the magnetic anisotropy fields of the top GaMnAs layer so that the sharp MR change observed at smaller $|H|$ is reproduced as much as possible, as shown in Supplementary Fig. S2a-d. Supplementary Fig. S2e shows the experimentally obtained MR characteristics at V_G = -3 V (thick black curves), and calculated MR characteristics (thin colored curves) with several ε/M of the bottom GaMnAs layer while other parameters needed for the calculations are fixed at the values used in the main manuscript. By choosing the best parameters so that the sharp MR change observed at larger $|H|$ is reproduced as much as possible, we can determine the parameters for the bottom GaMnAs layer.

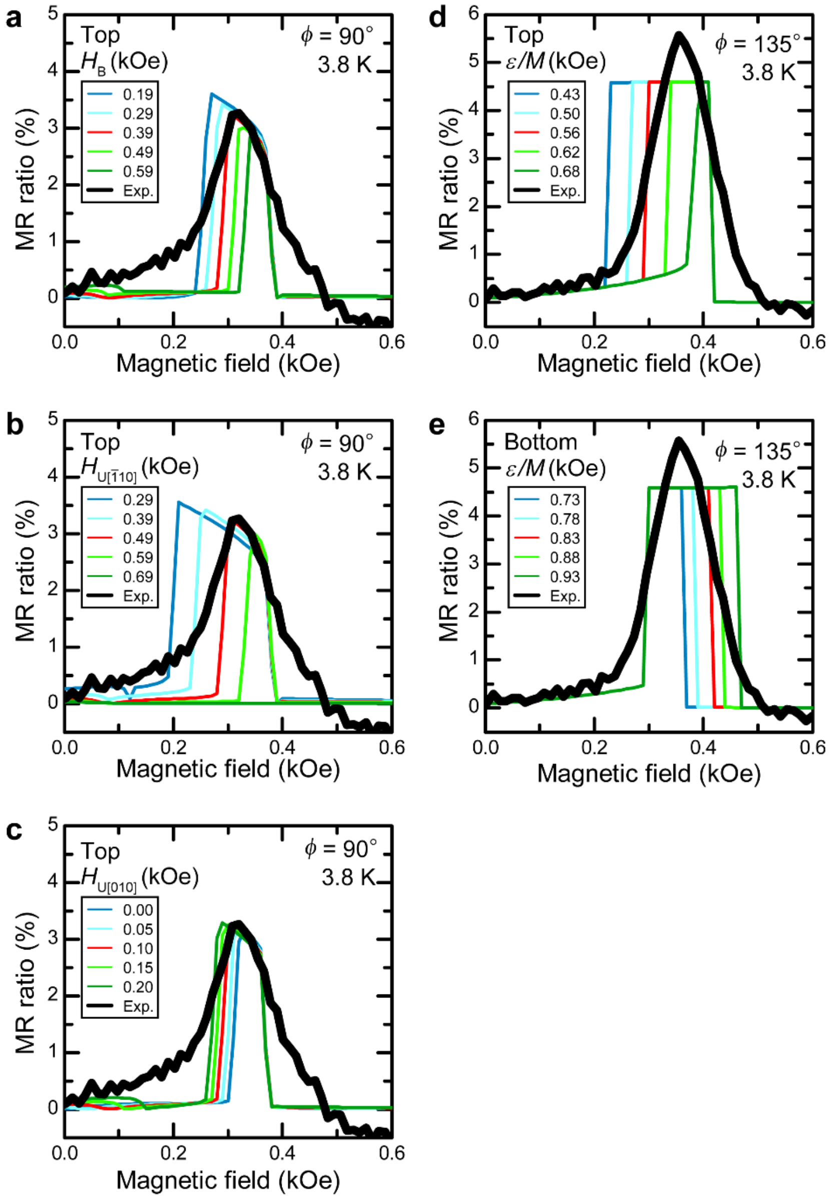

Supplementary Figure S2 | Experimental (black curves at V_{GS} = -3V) and calculated MR characteristics (colored curves) with various fitting parameters. In the calculation, H_B (**a**), $H_{U[\bar{1}10]}$ (**b**), $H_{U[010]}$ (**c**), and ε/M (**d**) of the top GaMnAs layer, and ε/M (**e**) of the bottom GaMnAs layer are varied, while parameters except for the varied one are fixed at the values used in the main manuscript in each graph. The calculated results explain the peak positions in experimental results well with the parameters used in the main manuscript (red curves in Supplementary Fig. S2).

Supplementary Note 3. Influence of the parasitic resistance in the device performance

The resistance of our device is dominated by the tunnel resistance at the GaAs barrier and the influence of the parasitic resistance is negligibly small as shown below. Supplementary Fig. S3 shows the resistance area product (RA) of the device prepared in the main manuscript (GaMnAs (9.2 nm)/ GaAs (11 nm)/ GaMnAs (3 nm)/ GaAs: Be (100 nm) on a p^+ GaAs (001) substrate) and that of the diode without the GaAs barrier layer which is composed of GaMnAs (10 nm)/ GaAs: Be (100 nm) on a p^+ GaAs (001) substrate. The RA of the diode without a GaAs barrier layer is ~10^{-3} – 10^{-4} times smaller than the device which has a GaAs barrier layer. Thus, the resistance originating from the GaAs: Be layer and the substrate is estimated to be only ~10^{-1} - 10^{-2}% of that of the whole MTJ. Because the Be concentration of the GaAs: Be layer is high (1×10^{18} cm^{-3}) and it is metallic, the influence of the gate electric field modulation of GaAs: Be is smaller than ~10^{-1} - 10^{-2}% of the total resistance of the MTJ. It is negligibly small in comparison with the change of the resistance obtained in our main manuscript (~20%).

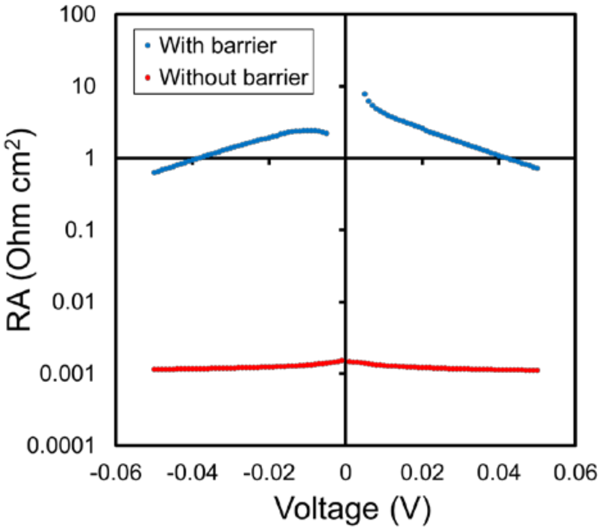

Supplementary Figure S3 | Influence of the parasitic resistance in the device performance. Bias voltage dependence of the RA of the diode composed of GaMnAs/GaAs/GaMnAs/GaAs:Be on a *p*+ GaAs (001) substrate, and GaMnAs/GaAs:Be on a p^+ GaAs (001) substrate at ~4 K.

Supplementary Note 4. Temperature dependence of magnetic anisotropy

We show that the Joule heating, which may be induced by the change in the current due to the electric field, is *not* the origin of the change in the anisotropy fields of the GaMnAs layers. We measured tunnel magnetoresistance (TMR) for a reference sample of a GaMnAs-based magnetic tunnel junction varying the (source-drain) bias voltage V and thus the tunneling current I. The reference sample is composed of $Ga_{0.94}Mn_{0.06}As$ (10 nm)/ GaAs (9 nm)/ $Ga_{0.94}Mn_{0.06}As$ (3 nm)/ GaAs: Be (100 nm) grown on a p^+GaAs (001) substrate by low temperature molecular beam epitaxy. We fabricated a simple cylindrical mesa diode with 100 μm in diameter. This device does not have a gate electrode. Here, we note that this reference sample is more sensitive to the influence of Joule heating than the sample used in the main manuscript. Because this reference sample has a thinner tunnel barrier than the sample prepared in the main manuscript, the generated heat per unit area becomes larger when the applied voltages are fixed. In addition, because the total surface area per unit volume of the reference sample is smaller than the sample prepared in the main manuscript which has an elongated shape, heat exchange from the surface is less efficient in this reference sample. The TMR measurements were performed in the same way as described in the main manuscript. Supplementary Fig. S4a-c shows the magnetic-field direction ϕ dependence of the MR ratio at V = 5 mV, 10 mV and 15 mV, respectively. The corresponding I which flows in the diode are 105 nA, 341 nA and 787 nA at V = 5 mV, 10 mV, and 15 mV when the magnetic field H is 0 kOe, respectively. Despite this large change in I (105 nA→787nA), the symmetry of the MR patterns looks very similar in Supplementary Fig. S4a-c. This means that the magnetic anisotropy is not changed by changing I or the Joule heating in this experiment. In our study shown in the main manuscript, the modulation ratio of the drain current by the gate electric field is only 20% at most (Fig. 2b in the main manuscript), which is much smaller than the change in I in this experiment (105 nA→787nA, 650%). Thus, we can conclude that the effect of the Joule heating is negligibly small for the change in the anisotropy fields.

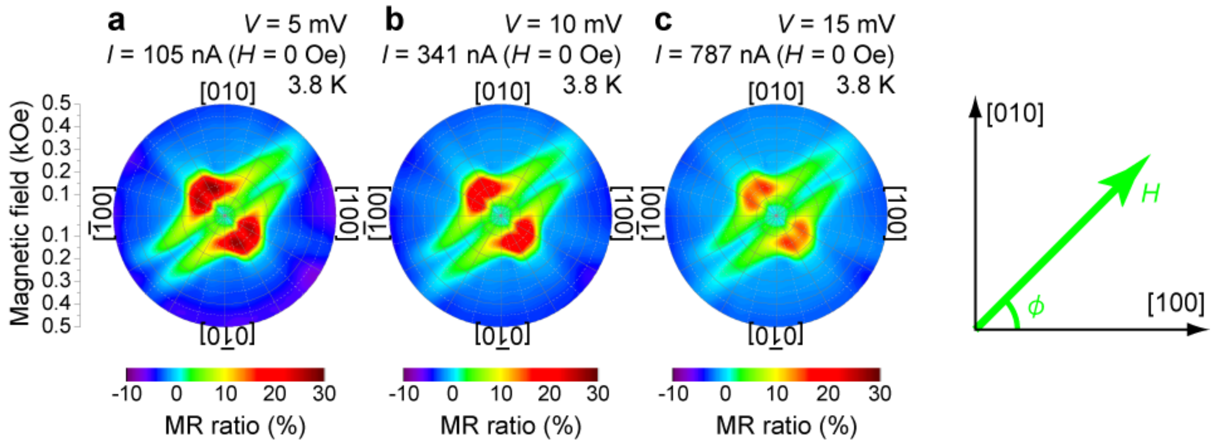

Supplementary Figure S4 | Temperature dependence of magnetic anisotropy. Experimental ϕ dependence of the MR at 5 mV (**a**), 10 mV (**b**), and 15 mV (**c**). The MR pattern looks very similar.
